# Presumed Ocular tuberculosis masquerading as autoimmune retinopathy

**DOI:** 10.1016/j.ajoc.2025.102296

**Published:** 2025-03-05

**Authors:** Si-meng Hou, Qian Liu, Xiao-hui Zhang, Xiao-yan Peng, Hui-yang Zeng

**Affiliations:** a Department of Retina, Beijing Tongren Eye Center, Beijing Tongren Hospital, Capital Medical University, Beijing, 100730, China; b Beijing Institute of Ophthalmology, Beijing Tongren Eye Center, Beijing Tongren Hospital, Capital Medical University, Beijing, 100730, China; c Beijing Key Laboratory of Ophthalmology & Visual Science, Beijing, China

**Keywords:** Ocular tuberculosis, Autoimmune retinopathy, Visual field constriction

## Abstract

**Purpose:**

To report a case of tuberculosis (TB) infection with an unusual posterior segment manifestation that mimicked autoimmune retinopathy (AIR).

**Observation:**

A 36-year-old male presented with blurred vision and constriction of visual field (VF) in both eyes for over 2 weeks. Multimodal imaging suggested he had AIR-like retinopathy; however, the initial local/systemic steroid treatment worsened his condition. He later tested positive for TB infection and received one month of anti-tuberculosis (ATT) monotherapy, followed by combined steroid treatment for an additional 5 months. He was followed up 12 months after treatment, demonstrating significantly improved visual function and restoration of macular anatomy.

**Conclusions:**

This case underscores the need to consider intraocular TB as a potential mimic of AIR, highlighting the importance of ruling out active infections before diagnosing AIR.

## Highlights

• Tuberculosis (TB) -related eye diseases show diverse manifestations, primarily affecting both the choroid and retina.
• We presented a rare case of presumed ocular TB mimicking autoimmune retinopathy (AIR), with outer retina damage.
• Rule out active TB infections before diagnosing AIR or using steroids and immunosuppressive treatments.

## 1 Introduction

Tuberculosis (TB)-associated posterior segment diseases of the eye exhibit a wide spectrum of manifestations, including tuberculoma, serpiginous choroiditis, multifocal choroiditis, and retinal vasculitis.^1^ Most of these phenotypes involve both the choroid and retinal vessels, whereas reports of damage limited to the outer retina are quite rare. Autoimmune retinopathy (AIR) is a group of immune-mediated diseases that can cause blindness, primarily affecting the outer retinal layers, including ellipsoid zone (EZ), external limiting membrane (ELM) and outer nuclear layer (ONL).^2^ Any apparent cause responsible for abnormalities in visual function in this disease entity, including infections, must be ruled out due to the treatment strategy that predominantly employs immunosuppressive agents. Here, we present a case of retinopathy due to presumed TB that mimicks features of AIR.

## 2 Case report

**Fig. 1:**
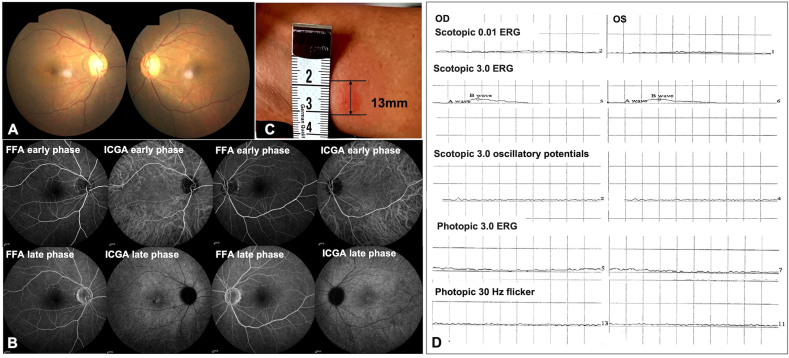
**Baseline images of the fundus, FA&ICGA, PPD, and ff-ERG of the patient**. (A) The fundus of both eyes showed slight pallor of the optic discs, mild arteriolar attenuation, and RPE mottling. (B) FFA and ICGA demonstrated unremarkable changes except for late-phase hyper-fluorescence in the posterior pole on ICGA without leakage. (C) PPD test showed a positive result with an induration size of 13mm. (D) The ff-ERG revealed nearly extinguished scotopic rod and photopic cone responses. FA: fluorescence angiography; ICGA: indocyanine green angiography; PPD: purified protein derivative; ff-ERG: full-field electroretinography. (For interpretation of the references to colour in this figure legend, the reader is referred to the Web version of this article.)

**Fig. 2:**
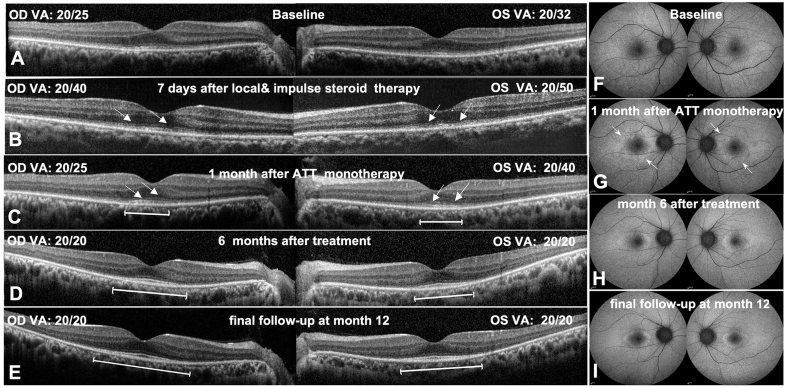
Changes in the retinal structure of the patient before and after treatment. (A) At baseline, SD-OCT revealed diffuse disruption of EZ and ELM with comparatively preserved fovea. (B) Deterioration of outer retinal damage, particularly the fovea, was observed after treatment with both local and systemic use of steroid (arrow). BCVA in both eyes also decreased compared to baseline. (C) Notable restoration of the EZ and ELM in the foveal area (arrow), along with significant increase in BCVA, occurred in both eyes following one month of ATT monotherapy. (D) Both the ELM and EZ within the fovea and para-fovea were fully restored following a combined therapy of 6 months of ATT and 3 months of steroid. The BCVA improved to 20/20 in both eyes. (E) At the final follow-up at month 12 after treatment, the well-maintained BCVA and outer retinal structure in the fovea and para-fovea were observed. Note the persisting loss of EZ and ELM in the extra-foveal region throughout the entire disease course, along with progressive thinning of the ONL (D&E). (F) At baseline, FAF revealed diffuse transmitting hyper-autofluorescence in the posterior pole in both eyes. (G) Following one month of ATT monotherapy, a HAF ring around the fovea on FAF appeared (indicated by the arrows), implying control of inflammation. (H&I) At month 6 and month 12 after treatment, FAF showed a clearly defined HAF ring in both eyes. EZ: ellipsoid zone; ELM: external limiting membrane; BCVA: best-corrected visual acuity; ATT: anti-tuberculosis; ONL: outer nuclear layer; FAF: fundus autofluorescence; HAF: hyper-autofluorescence.

**Fig. 3:**
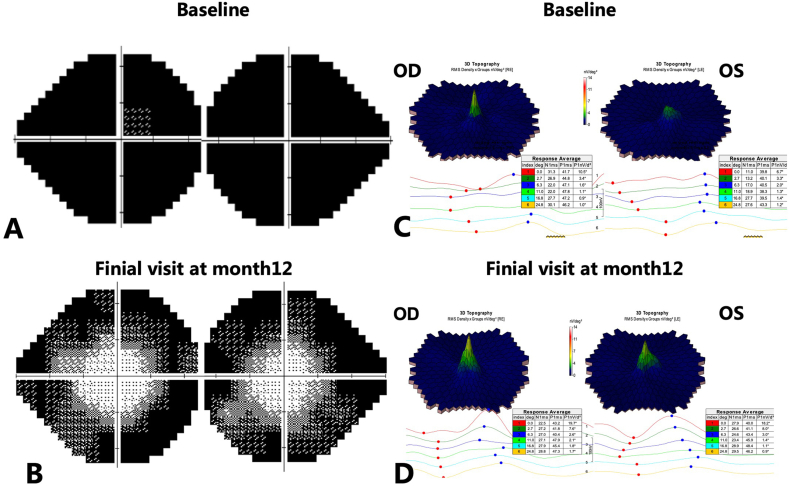
Changes in visual function of the patient before and after treatment. (A&B) The baseline VF showed severe constriction in both eyes, with a loss of the central island in the left eye. Significant expansion of central VF was observed in both eyes 12 months after treatment. (C&D) The baseline mf-ERG showed a widespread reduction in amplitude, with a greater decrease in the left eye. However, at the 12-month follow-up, a notable increase in amplitude was observed, especially in the left eye. VF: visual field; mf-ERG: multifocal electroretinography.

A 36-year-old male presented to our retinal clinic with a complaint of constriction of the visual field (VF) in both eyes over the last two weeks. He reported a large alcohol intake for three consecutive days at the time visual symptoms started. Two weeks prior to onset of the symptoms, the patient suffered from a COVID-19 infection, experiencing a high fever (39 °C) for two days. His review of systems was otherwise unremarkable, with no indications of autoimmune diseases, malignancy, or other significant medical history. He also denied any family history of acquired or inherited eye diseases, particularly retinitis pigmentosa (RP). Best corrected visual acuity (BCVA) was 20/25 in the right eye (OD) and 20/32 in the left eye (OS). Intraocular pressure was normal in both eyes (OU). Slit-lamp examination showed no signs of inflammation in either the anterior segment or vitreous. Fundoscopy OU revealed slight pallor of optic discs, mild arteriolar attenuation, and retinal pigment epithelium (RPE) mottling (Fig. 1A). Spectral domain optical coherence tomography (SD-OCT) (Fig. 2A) indicated severe extrafoveal outer retinal damage, including extensive loss of the ELM, EZ and interdigitation zone (IZ). Although the foveal area was comparatively preserved, there was still evidence of patchy EZ attenuation. Fundus autofluorescence (FAF) OU revealed diffuse transmitting hyper-autofluorescence in the posterior pole (Fig. 2F). Fluorescein angiography (FA) OU showed staining of the optic discs both eyes, while otherwise normal. Indocyanine green angiography (ICGA) showed extensive hyper-fluorescence in the late frames, with no leakage (Fig. 1B). Humphrey visual field (HVF) testing showed severe field constriction in both eyes, with a loss of the central island in the left eye (Fig. 3A), corresponding to the impairment of outer segments on OCT. Full-field electroretinography (ffERG) OU revealed nearly extinguished scotopic rod and photopic cone responses (Fig. 1D). Multifocal electroretinography (mf-ERG) showed a diffuse reduction in amplitude, with a greater decrease noted in the left eye (Fig. 3C).

We initially diagnosed him with presumed-AIR or AIR-like retinopathy based on the above findings and had the patient undergo a chest computed tomography (CT) scan, a whole-body positron emission tomography (PET) scan and a comprehensive laboratory work-up, which included a complete blood count, blood biochemistry, human immunodeficiency virus (HIV) antibody test, T-spot.TB, toxoplasma titers, fluorescent treponemal antibody absorption (FTA-ABS), hepatitis virus serology, thyroid-stimulating hormone, anti-nuclear antibody, anti-neutrophil cytoplasmic antibodies, rheumatoid factor and C-reactive protein. Additionally, Western blot (WB) assay was performed to detect the presence of anti-retinal antibodies (ARAs), targeting antibodies against recoverin, α-enolase, carbonic anhydrase II antibody (CA-II), heat shock protein (HSP)-60, aldolase-C, collapsing response mediator protein (CRMP)-5, arrestin, phosphate dehydrogenase gene (GADPH) and tubby-like protein (TULP)-1. Two days later, the chest CT scan and most laboratory test results returned normal, except for the TB assay (T-spot, interferon gamma release assays [IGRA]), the ARAs test, and the PET scan, which were still pending. Based on a low suspicion of TB, we administered retrobulbar injections of dexamethasone 5 mg in both eyes as a diagnostic treatment for presumed AIR. Two days after the injection, he reported no improvement and requested for further treatment when we contacted him by phone, as he had returned to his hometown. We then recommended a five-day intravenous course of 500 mg methylprednisolone as pulse therapy at a nearby hospital. After the treatment, he returned to our facility for a follow-up. The patient's visual acuity decreased from 20/25 to 20/40 OD and from 20/32 to 20/50 OS with more outer segment disruption observed in the fovea of both eyes (Fig. 2B). Meanwhile, an unexpectedly positive result from the IGRA was obtained (82.1 pg/ml), indicating a level ten times higher than normal range of 0–14 pg/ml. The PET scan confirmed no systemic malignancy. Further investigation revealed that the patient had experienced night sweats for over four years and had not received Bacillus Calmette-Guerin (BCG) immunization. Additionally, his aunt had a medical history of bone TB. The patient was subsequently administered a purified protein derivative (PPD) test at another institution, which yielded a positive result with induration size of 13mm (Fig. 1C). This led us to believe that the TB infection was the cause of the worsening retinal conditions during corticosteroid treatment.

The patient was then advised to discontinue steroid use and referred to the department of infectious disease, where he was prescribed a traditional ATT regimen consisting of rifampicin, isoniazid, and pyrazinamide. After a week of ATT treatment, the patient exhibited a slight improvement in vision, with 20/30 OD and 20/40 OS, and OCT findings indicated the EZ was beginning to restore (data not shown). These results suggested a positive response to the ATT therapy. In the first month following ATT monotherapy, the foveal EZ showed further restoration, and the visual acuity continued to improve (Fig. 2C), accompanied by the appearance of a hyper-autofluorescence (HAF) ring around fovea on FAF, indicating control of inflammation (Fig. 2G). Meanwhile, the patient's serum tested positive for antibody against α-enolase on WB assay. At this time point, presumed ocular tuberculosis masquerading as autoimmune retinopathy was diagnosed. We administered a retrobulbar triamcinolone acetonide (TA) injection to the patient and prescribed a weekly tapering of the 40 mg/day oral corticosteroid dosage. In the second month following ATT treatment, the EZ and ELM in the fovea appeared more distinct and integrated, with BCVA increasing to 20/20 OD and 20/30 OS. The patient's subjective visual function significantly improved during this time. However, he admitted that he refrained from using oral steroids due to concerns that the condition might deteriorate while on the medication, as it had during the initial round of methylprednisolone treatment. We reassured him of the effectiveness of the systemic steroid in combination with ATT treatment and administered another retrobulbar TA. Subsequently, he began taking oral prednisone as directed. After 6 months of ATT medication and 3 months of oral steroids, the patient's BCVA improved to 20/20 OU and both the EZ and ELM within the fovea and parafovea showed full restoration (Fig. 2D), although there was extensive loss of extrafoveal EZ and ELM. At this stage, FAF revealed a clearly defined HAF ring that had partially repaired, indicating that the inflammation was fully under control (Fig. 2H). From this point forward, the patient was placed under observation. At the final visit, 12 months after treatment, we observed well-maintained BCVA, restored foveal and parafoveal EZ, and a typical HAF ring (Fig. 2E and I). However, the extra-foveal region consistently showed a lack of EZ and ELM, as well as thinning of the ONL (Fig. 2D and E). In line with the anatomic restoration of the fovea, the VF demonstrated a marked expansion of the central island OU (Fig. 3A and B). Additionally, mf-ERG indicated a significant increase in amplitude, particularly in the left eye compared to baseline (Fig. 3C and D), implying an improvement in macular function. However, ff-ERG did not reveal any significant improvement in both rod and cone responses (data not shown), possibly due to the extensive loss of the extrafoveal outer retinal segment.

## 3 Discussion

China has a high TB burden with prevalence of 7.36 %, second only to India.^3^ Therefore, in all cases of inflammatory disorders affecting the posterior segment of the eye, ocular tuberculosis (OTB) should be highly suspected. The chorioretinopathy associated with TB can arise from either a direct bacterial infection or an immune response to bacterial antigens.^1^^,^^3^ OTB can present in various forms, with tuberculous posterior uveitis being the most common variant.^1^^,^^3^, ^4^, ^5^ In this report, we described a case of presumed OTB masquerading as AIR, a rare retinal degenerative condition, with a one-year follow-up after treatment.

The diagnosis of OTB in this patient was confirmed through positive results from both the IGRA and PPD test. Recent studies indicate that interpretation of IGRA in conjunction with PPD findings increase both sensitivity and specificity for diagnosing TB.^6^ Strong evidence linking TB infection to retinal disease in this case includes significant improvement in visual function and macular structure after one month of ATT treatment ALONE, while the initial use of steroids worsened the condition. The absence of TB vaccination, a family history of TB, and night sweats further indicated TB infection and activation. We speculated that, in this patient, heavy alcohol consumption or high fever caused by a COVID-19 viral infection may be risk factors for the progression of latent TB to active OTB by compromising immune function.^6^

Although outer retinal involvement such as those seen in multiple evanescent white dot syndrome (MEWDS)^4^ and acute posterior multifocal placoid pigment epitheliopathy (APMPPE)^5^ are linked to an antecedent viral illness, few cases have also been reported with TB infection. Recently, Martins Melo et al. reported a rare case of suspected ocular TB presenting with bilateral pseudo-retinitis pigmentosa.^7^ This patient, suffering from pulmonary TB, experienced one-year course of eye disease and showed improvement in FA findings with ATT monotherapy, although visual function remained unchanged. Our case appeared similar to this phenotype, but with less RPE mottling, possibly due to a shorter disease duration. The diagnosis of presumed OTB masquerading as AIR was based on the evidence of TB infection, the effect of initial ATT monotherapy, and clinical features of AIR, such as sub-normal fundus, binocular outer retinal damage on OCT, HAF ring, visual field constriction, flat ERG response and positivity of serum ARAs.^2^ However, this diagnosis must be differentiated from AIR, as all apparent causes, including active infections, need to be ruled out.^2^ Although the production of ARAs may arise from molecular mimicry between retinal antigens and bacterial or viral proteins, AIR is considered to be an immune-mediated retinopathy rather than a direct bacterial attack. Therefore, immunosuppression is used to treat AIR instead of targeting the potential microbe.^2^^,^^8^ On the other hand, despite many AIR patients showing poor response to steroid treatment,^2^^,^^8^ if a patient truly has AIR, their condition should not worsen shortly after starting treatment with steroid, as demonstrated in our patient's initial treatment. Notable and rapid improvement, as observed in our patient, is rarely seen in AIR cases.^2^^,^^8^ The presence of serum ARA, including α-enolase, is not specific to AIR and can also be observed in both infectious and non-infectious uveitis.^2^

Additionally, we need to differentiate other binocular outer retinal inflammatory and degenerative disorders, particularly those associated with COVID-19. Although there may be a temporal connection, we believe that the high fever caused by COVID-19 virus infection likely triggered latent TB rather than causing eye disease. COVID-19-associated outer retinal disorders, including APMPPE,^9^ multiple focal choroiditis (MFC),^10^ punctate inner choroidopathy (PIC),^11^ and serpiginous choroiditis(SC),^12^ generally respond well to steroid treatment or recover spontaneously. Moreover, TB infection has been confirmed in this patient. Other potential etiologies, such as acute zonal occult outer retinopathy (AZOOR), can be ruled out due to the patient's extensive, symmetrical lesions exacerbated by steroids. Syphilitic outer retinopathy, retinitis pigmentosa sine pigmento (RPSP), and acute alcoholic toxic retinopathy can also be excluded through lab testing and treatment response.

Throughout the follow-up, the patient exhibited persistent loss of the EZ, ELM, along with gradual thinning of the ONL beyond the macula. Prior studies^9^, ^10^, ^11^, ^12^, ^13^ suggest *that minor to moderate damage to the EZ and ELM*, *as observed in this patient's fovea, may have repair* potential when *underlying causes, such as inflammation and infection,* are treated*. However, extensive damage,* as noted *in the extrafoveal* region of this patient, is challenging to repair.^14^^,^^15^ *ONL atrophy is believed to result from extensive loss of EZ and ELM* because EZ damage impairs mitochondrial function in photoreceptors, causing cell death, while ELM damage disrupts structural support, resulting in cell disarray and atrophy.^14^, ^15^, ^16^Despite this, the restoration of macular function and structural integrity was evident under aggressive treatment.

## 4 Conclusion

Our report highlights that OTB remains a challenging diagnosis with diverse atypical manifestations, such as AIR-like retinopathy. We have detailed the clinical course, treatment response, and visual outcome of this phenotypes over a long-term follow-up. Infections such as TB must be ruled out prior to the diagnosis of AIR or the initiation of steroids and immunosuppressive medications, as the elimination of the etiological factor is the primary therapeutic strategy for OTB.

## CRediT authorship contribution statement

**Si-meng Hou:** Writing – original draft, Methodology, Investigation, Data curation. **Qian Liu:** Methodology, Investigation, Data curation. **Xiao-hui Zhang:** Resources, Investigation, Data curation. **Xiao-yan Peng:** Project administration, Methodology, Conceptualization. **Hui-yang Zeng:** Writing – review & editing, Project administration, Methodology, Funding acquisition, Conceptualization.

## Patient consent

The patient consented to publication of the case orally. This report does not contain any personal identifying information.

## Funding

This work was supported by Beijing Municipal Natural Science Foundation [7192034, H.Y.Z], Key research program of the Beijing Institute of Ophthalmology (H.Y.Z.); the National Natural Science Foundation of China (81100675)

## Declaration of competing interest

The author is not an Editorial Board Member/Editor-in-Chief/Associate Editor/Guest Editor for *[American Journal of Ophthalmology Case Reports]* and was not involved in the editorial review or the decision to publish this article.

The authors declare the following financial interests/personal relationships which may be considered as potential competing interests: The author had no known competing financial interests or personal relationships that could have appeared to influence the work reported in this paper.
